# Effect of nodal status on clinical outcomes of triple-negative breast cancer: a population-based study using the SEER 18 database

**DOI:** 10.18632/oncotarget.9432

**Published:** 2016-05-18

**Authors:** Xiao-Xiao Wang, Yi-Zhou Jiang, Jun-Jing Li, Chuan-Gui Song, Zhi-Ming Shao

**Affiliations:** ^1^ Department of Breast Surgery, Affiliated Union Hospital, Fujian Medical University, Fuzhou, China; ^2^ Department of Breast Surgery, Key Laboratory of Breast Cancer, Fudan University Shanghai Cancer Center, Shanghai Medical College, Fudan University, Shanghai, China

**Keywords:** nodal status, tumor size, triple-negative breast cancer, breast cancer-specific survival, overall survival

## Abstract

Triple-negative breast cancer (TNBC) is an aggressive malignancy with a poor prognosis. Data from the Surveillance, Epidemiology and End Results database (2010–2012) were used to identify 10,771 patients with TNBC, and we assessed the effects of lymph node (LN) status on breast cancer-specific survival (BCSS) and overall survival (OS). In our study, a Kaplan-Meier plot showed that LN-negative patients (N0) had better survival outcomes than LN-positive patients and that patients with ≥10 positive LNs (N3) exhibited the worst survival outcomes regardless of tumor size. A pairwise comparison showed no difference in survival outcomes among each group stratified by tumor size. Further, for LN-positive patients with a tumor size ≤2 cm (T1) or >5 cm (T3), there were similar outcomes between patients with one to three LNs (N1) and those with four to nine LNs (N2), whereas N1 patients experienced significantly better survival outcomes than N3 patients (P<0.001). Therefore, ten metastatic lymph nodes was the cut-off value for poor prognosis. Nevertheless, for patients with a tumor size of 2-5 cm (T2), the extent of LN involvement contributed prognostic value to OS but not BCSS. In summary, we found that nodal status and tumor size exhibited distinct interaction patterns for predicting the outcomes of TNBC. These results provide deeper insight into the prognostic value of nodal status in TNBC.

## INTRODUCTION

Triple-negative breast cancer (TNBC), defined as a tumor that lacks expression of the oestrogen receptor (ER), the progesterone receptor (PR), and human epidermal growth factor receptor 2 (HER2), accounts for 15–20% of all breast cancer cases [1]. TNBC is associated with younger age at diagnosis, African-American race, higher histological grade, larger size, more advanced disease stage and a tendency towards local and visceral metastases rather than bone metastases [2–6]. TNBC is characterized by high invasiveness, poor prognosis, and an early peak of recurrence within the first 3 years, as well as a higher 5-year mortality rate than other breast cancer subtypes [7, 8].

Given the poor prognosis of TNBC, cancer-related outcomes must be estimated accurately. Several factors responsible for the poor clinical outcomes observed in TNBC, including age, race, grade, tumor size, lymph node status, and distant metastasis, have been studied. Among these factors, tumor size, lymph node status, and distant metastasis serve as important prognostic determinants and constitute the American Joint Committee on Cancer (AJCC) staging system [9]. As such, lymph node status has great clinical significance in guiding the treatment of breast cancer. Accumulating studies have focused on nodal status as one of the most crucial prognostic factors in breast cancer patients [10–12]. For instance, nodal status has been associated with overall prognosis [13, 14]. However, other studies did not confirm the prognostic significance of nodal status in TNBC [15, 16]. Arvold *et al.* [17] demonstrated that greater numbers of positive lymph nodes and tumor size were not significantly associated with increased risk of locoregional recurrence in their TNBC cohort. In addition, Hernandez-Aya *et al.* [18] elucidated that TNBC patients with positive lymph node status experienced worse overall survival (OS) and relapse-free survival (RFS) but that the prognosis of these patients may not be affected by the number of positive lymph nodes. Based on small numbers of patients and different populations, investigators have arrived at discordant conclusions. From these relevant studies, we consider that the prognostic value of nodal status continues to remain uncertain and controversial. Therefore, it is necessary to further elucidate the relationship between nodal status and the prognosis of TNBC patients in a larger cohort.

This study was designed to investigate the effects of lymph node status on breast cancer-specific survival (BCSS) and OS among TNBC patients by utilising population-based Surveillance, Epidemiology, and End Results (SEER) data to confirm whether nodal status has prognostic significance.

## RESULTS

### Demographic and clinical characteristics of the study population

As illustrated in Table 1, a total of 10,771 patients met the eligibility criteria for our study. Among these patients, 69.5% (n=7481) of the patients were classified as N0 (lymph node negative), 21.9% (n=2355) as N1 (one to three positive lymph nodes), 5.2% (n=562) as N2 (four to nine positive lymph nodes), and 3.5% (n=373) as N3 (≥10 positive lymph nodes). Compared with lymph node-negative patients, patients with nodal involvement tended to be younger (the median age of N0 patients was 57 years; P<0.001), presented with a higher histological grade (P<0.001), and were more likely to have a greater tumor size. Furthermore, as the number of positive lymph nodes increased, the tumor size also increased. In addition, lymph node-positive patients underwent mastectomy more frequently than N0 patients (42.5%, 54.1%, 70.3%, and 71.0% for N0, N1, N2, and N3 patients, respectively; P<0.001).

**Table 1:** Demographic and clinical characteristics of the study population

Characteristics	N0 (n=7481)		N1 (n=2355)		N2 (n=562)		N3 (n=373)		P^c^
	No.	%	No.	%	No.	%	No.	%	
**Median follow-up duration (months) (IQR)**	16 (8-26)		16 (8-25)		17 (10-25)		14 (7-22.5)		
**Age (years)**									**<0.001**
**Median**	57		53		54		53		
**<50**	2165	28.9	941	40.0	205	36.5	138	37.0	
**≥50**	5316	71.1	1414	60.0	357	63.5	235	63.0	
**Race**									**0.003**
**White**	5529	73.9	1649	70.0	409	72.8	258	69.2	
**Black**	1422	19.0	525	22.3	121	21.5	87	23.3	
**Other^a^**	530	7.1	181	7.7	32	5.7	28	7.5	
**Marital status**									0.075
**Married**	4630	61.9	1443	61.3	327	58.2	211	56.6	
**Not married^b^**	2851	38.1	912	38.7	235	41.8	162	43.4	
**Laterality**									0.143
**Left**	3882	51.9	1211	51.4	313	55.7	180	48.3	
**Right**	3599	48.1	1144	48.6	249	44.3	193	51.7	
**Histological type**									**<0.001**
**Infiltrating duct carcinoma**	7397	98.9	2334	99.1	555	98.8	360	96.5	
**Lobular carcinoma**	84	1.1	21	0.9	7	1.2	13	3.5	
**Histological grade**									**<0.001**
**I/II**	1532	20.5	306	13.0	71	12.6	54	14.5	
**III**	5949	79.5	2049	87.0	491	87.4	319	85.5	
**Tumor size (cm)**									**<0.001**
**≤2**	4343	58.1	747	31.7	130	23.1	72	19.3	
**>2 and ≤5**	2825	37.8	1294	54.9	327	58.2	194	52.0	
**>5**	313	4.2	314	13.3	105	18.7	107	28.7	
**Type of surgery**									**<0.001**
**None**	196	2.6	135	5.7	15	2.7	15	4.0	
**Breast-conserving surgery**	4102	54.8	946	40.2	152	27.0	93	24.9	
**Mastectomy**	3183	42.5	1274	54.1	395	70.3	265	71.0	
**Radiation therapy**									**<0.001**
**No**	4039	54.0	1090	46.3	196	34.9	235	63.0	
**Yes**	3442	46.0	1265	53.7	366	65.1	138	37.0	

Abbreviation: IQR, interquartile range.

a Other includes American Indian/native Alaskan and Asian/Pacific Islander.

b Not married includes divorced, separated, single (never married), unmarried or domestic partner and widowed.

c P values were calculated among all groups using a Chi-squared test, and bold type indicates significance.

### Survival estimates and pairwise comparisons according to tumor size and lymph node status

**Figure 1:**
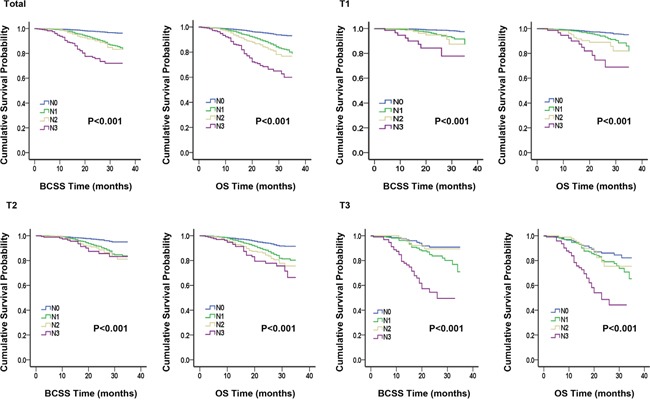
Kaplan-Meier plot and log-rank tests comparing breast cancer-specific survival (BCSS) and overall survival (OS) between different nodal status stages according to tumor size classification

A Kaplan-Meier plot and log-rank tests were used to compare BCSS and OS between different subgroups according to tumor size classifications, and the results are listed in Table 2 and Figure 1. There were 337 deaths due to breast cancer and 547 deaths due to all causes. For each tumor size category, the Kaplan–Meier survival curves of different groups stratified by lymph node status were distinctly separated. Significant differences in survival outcomes were observed between N0 patients and N1–N3 patients. This result indicated that nodal status was associated with BCSS and OS (both P<0.001). Regardless of the tumor size, the N0 group exhibited better survival outcomes and the N3 group exhibited worse survival outcomes.

**Table 2:** Estimates of BCSS and OS and pairwise comparisons according to tumor size and lymph node status

Tumor size/nodal status	No. of patients	BCSS				OS			
		No. of events	Overall P^a^	Pairwise	Adjusted P^b^	No. of events	Overall P^a^	Pairwise	Adjusted P^b^
**Total**	10771	337	**<0.001**	N0 v N1	0.133	547	**<0.001**	**N0 v N1**	**0.011**
**N0**	7481	115		N0 v N2	0.654	234		N0 v N2	0.785
**N1**	2355	130		N0 v N3	0.377	177		N0 v N3	0.999
**N2**	562	38		N1 v N2	1.000	63		N1 v N2	0.916
**N3**	373	54		**N1 v N3**	**0.002**	73		N1 v N3	0.054
				N2 v N3	0.053			N2 v N3	0.729
**T1**			**<0.001**	N0 v N1	0.984		**<0.001**	N0 v N1	0.114
**Total**	5292	76		N0 v N2	0.944	151		N0 v N2	0.999
**N0**	4343	37		N0 v N3	0.865	93		N0 v N3	1.000
**N1**	747	25		N1 v N2	0.998	35		N1 v N2	0.874
**N2**	130	6		N1 v N3	0.610	12		N1 v N3	0.836
**N3**	72	8		N2 v N3	0.592	11		N2 v N3	1.000
**T2**			**<0.001**	N0 v N1	0.138		**<0.001**	N0 v N1	0.248
**Total**	4640	178		N0 v N2	0.678	280		N0 v N2	0.963
**N0**	2825	62		N0 v N3	1.000	112		N0 v N3	1.000
**N1**	1294	75		N1 v N2	1.000	102		N1 v N2	0.986
**N2**	327	26		N1 v N3	0.542	39		N1 v N3	0.874
**N3**	194	15		N2 v N3	0.829	27		N2 v N3	0.999
**T3**			**<0.001**	N0 v N1	0.841		**<0.001**	N0 v N1	0.854
**Total**	839	83		N0 v N2	1.000	116		N0 v N2	0.931
**N0**	313	16		N0 v N3	0.987	29		N0 v N3	0.993
**N1**	314	30		N1 v N2	0.993	40		N1 v N2	1.000
**N2**	105	6		N1 v N3	0.190	12		N1 v N3	0.375
**N3**	107	31		N2 v N3	0.987	35		N2 v N3	0.670

a Overall P was calculated using the Kaplan-Meier method and the log-rank test, and bold type indicates significance.

b Adjusted P was calculated using Sidak pairwise comparisons, and bold type indicates significance.

We conducted six Sidak-adjusted pairwise comparisons of BCSS and OS between the different lymph node status subgroups. The adjusted P values from the pairwise comparisons and the significance of the results are listed in Table 2. In the total cohort, significant differences in prognosis were observed between the N1 and N0 groups and between the N1 and N3 groups. However, no differences in BCSS or OS between the groups stratified by tumor size were observed.

### Analyses of outcome-related factors using Cox proportional hazard regression models

The results of the analyses of BCSS and OS using univariate and multivariate Cox proportional hazard regression models are shown in Supplementary Table S1 and Table 3, respectively. According to the univariate analysis shown in Supplementary Table S1, compared with patients with a negative lymph node status, patients with a positive lymph node status exhibited significantly worse BCSS (hazard ratio (HR), 3.692; 95% confidence interval (CI), 2.872 to 4.745 for N1 disease; HR, 4.300; 95% CI, 2.979 to 6.205 for N2 disease; and HR, 11.377; 95% CI, 8.231 to 15.725 for N3 disease; P<0.001) and OS (HR, 2.461; 95% CI, 2.025 to 2.992 for N1 disease; HR, 3.493; 95% CI, 2.645 to 4.614 for N2 disease; and HR, 7.440; 95% CI, 5.719 to 9.678 for N3 disease; P<0.001). Moreover, as the extent of lymph node involvement increased from N1 to N3, the HRs of BCSS and OS increased. The same results were observed based on multivariate analysis. In addition, married status, lower histological grade, a tumor size of ≤2 cm, and receipt of surgery and radiation therapy were independently associated with increased BCSS and OS according to univariate and multivariate analyses.

**Table 3:** Multivariate Cox proportional hazard model for assessing outcome-related factors

Variable	BCSS			OS		
	HR	95% CI	P^c^	HR	95% CI	P^c^
**Age (years)**						
**≥50**	1			1		
**<50**	0.974	0.778-1.218	0.815	0.773	0.643-0.929	**0.006**
**Race**			0.144			**0.029**
**White**	1			1		
**Black**	1.051	0.811-1.362	0.709	1.047	0.855-1.283	0.655
**Other^a^**	0.606	0.359-1.023	0.061	0.569	0.370-0.876	**0.010**
**Marital status**						
**Married**	1			1		
**Not married^b^**	1.144	0.915-1.427	0.234	1.269	1.067-1.508	**0.007**
**Grade**						
**III**	1			1		
**I/II**	0.697	0.490-0.994	**0.046**	0.882	0.690-1.128	0.316
**Tumor size**			**<0.001**			**<0.001**
**T1**	1			1		
**T2**	1.884	1.427-2.487	**<0.001**	1.644	1.338-2.019	**<0.001**
**T3**	3.735	2.653-5.259	**<0.001**	3.032	2.322-3.959	**<0.001**
**Lymph node status**			**<0.001**			**<0.001**
**N0**	1			1		
**N1**	2.836	2.182-3.686	**<0.001**	2.024	1.650-2.482	**<0.001**
**N2**	3.311	2.260-4.850	**<0.001**	2.940	2.199-3.931	**<0.001**
**N3**	7.725	5.464-10.922	**<0.001**	5.599	4.228-7.415	**<0.001**
**Type of surgery**			**<0.001**			**<0.001**
**Breast-conserving surgery**	1			1		
**Mastectomy**	1.167	0.902-1.510	0.240	1.208	0.986-1.480	0.069
**None**	2.810	1.800-4.387	**<0.001**	3.067	2.179-4.317	**<0.001**
**Radiation therapy**						
**No**	1			1		
**Yes**	0.659	0.520-0.835	**0.001**	0.582	0.481-0.704	**<0.001**

Abbreviation: CI, confidence interval.

a Other includes American Indian/native Alaskan and Asian/Pacific Islander.

b Not married includes divorced, separated, single (never married), unmarried or domestic partner and widowed.

c P values were adjusted using a multivariate Cox proportional hazard regression model including all factors, as categorized in Table 3, and bold type indicates significance.

### BCSS and OS in tumor size categories stratified by lymph node involvement

Considering the above confounding factors affecting TNBC outcomes, we further evaluated the BCSS and OS of different tumor-size groups stratified by the number of involved lymph nodes after adjusting for potential confounding factors. Among all of the patients, there was a significance difference in BCSS and OS between N0 patients and N1, N2, and N3 patients (P<0.001). N1 was used as a reference, and multivariate Cox proportional hazard regression models of BCSS and OS in each of the three tumor-size groups stratified by nodal status are shown in Table 4. For example, for patients with a tumor size ≤2 cm (T1), there were significant differences in survival outcomes between the N1 subgroup and the N0 and N3 subgroups. Those with N1 lymph node status experienced significantly better BCSS and OS than those with N3 lymph node status (HR, 4.142; 95% CI, 1.843-9.305; P=0.001 for BCSS; HR, 4.046; 95% CI, 2.033-8.049; P<0.001 for OS). However, those with N1 lymph node status experienced worse BCSS and OS than those with N0 lymph node status (HR, 0.258; 95% CI, 0.155-0.430 for BCSS; HR, 0.462; 95% CI, 0.312-0.683 for OS; both P<0.001). In addition, there was no significant difference in the HRs between the N1 and the N2 subgroups. Similar results were observed in patients with a tumor size >5 cm (T3). However, in the group of patients with a tumor size of 2-5 cm (T2), these analogous relationships were no longer apparent. Instead, the number of positive lymph nodes was inversely correlated with OS (HR=1.548 and 2.090 for N2 and N3, respectively). In contrast, the number of positive lymph nodes was not associated with BCSS. There was a significant difference in BCSS between the N1 and N0 groups (P<0.001) but not between the N1 group and either the N2 or N3 group (P=0.203 and P=0.114, respectively).

**Table 4:** Multivariate Cox proportional hazard model assessing the effect of tumor size stratified by the extent of lymph node involvement

Tumor size/nodal status	BCSS			OS		
	HR	95% CI	P^a^	HR	95% CI	P^a^
**Total**			**<0.001**			**<0.001**
**N1**	1			1		
**N0**	0.296	0.229-0.383	**<0.001**	0.427	0.349-0.521	**<0.001**
**N2**	1.192	0.827-1.718	0.346	1.148	1.110-1.985	**<0.001**
**N3**	3.193	2.314-4.406	**<0.001**	3.203	2.430-4.221	**<0.001**
**T1**			**<0.001**			**<0.001**
**N1**	1			1		
**N0**	0.258	0.155-0.430	**<0.001**	0.462	0.312-0.683	**<0.001**
**N2**	1.098	0.442-2.729	0.841	1.473	0.754-2.878	0.257
**N3**	4.142	1.843-9.305	**<0.001**	4.046	2.033-8.049	**<0.001**
**T2**			**<0.001**			**<0.001**
**N1**	1			1		
**N0**	0.370	0.262-0.522	**<0.001**	0.487	0.370-0.641	**<0.001**
**N2**	1.098	0.897-2.764	0.203	1.548	1.062-2.255	**0.023**
**N3**	4.142	0.552-1.785	0.114	2.090	1.358-3.216	**0.001**
**T3**			**<0.001**			**<0.001**
**N1**	1			1		
**N0**	0.480	0.259-0.890	**0.020**	0.605	0.372-0.985	**0.043**
**N2**	0.651	0.268-1.583	0.344	0.942	0.490-1.814	0.859
**N3**	3.514	2.098-5.884	**<0.001**	3.026	1.899-4.822	**<0.001**

Abbreviation: CI, confidence interval.

a P values were adjusted using a multivariate Cox proportional hazard regression model including age, race, marital status, grade, type of surgery, and radiation therapy, and bold type indicates significance.

## DISCUSSION

In this large cohort of patients, we sought to determine the interaction effect of lymph node status and tumor size on clinical outcomes among TNBC patients utilising population-based SEER data. Our findings indicated that the lymph node-positive group had a larger tumor size than the lymph node-negative group. Additionally, our results reinforced the concept reported in previous studies that as the number of positive lymph nodes increased, the tumor size also increased [18, 19]. However, when we conducted pairwise comparisons using the Sidak adjustment method, significant differences in prognosis were observed only between N1 and both N0 and N3. Further, to minimize the influence of tumor size on prognosis, we evaluated BCSS and OS between nodal status groups stratified by tumor size using a multivariate Cox proportional hazard regression model. Importantly, among the T1 and T3 cohorts, the N1 subgroups exhibited similar BCSS and OS to the N2 subgroups, whereas the N3 subgroups tended to experience worse outcomes than the N1 subgroups. Therefore, ten metastatic lymph nodes served as the cut-off value for poor prognosis. Furthermore, in the T2 group, the number of positive lymph nodes contributed prognostic value to OS, and as the number of lymph nodes increased, OS decreased. However, a significant difference in BCSS was observed between N1 and N0 but not between N1 and either N2 or N3.

Our results indicated that patients with lymph-node negative TNBC had a clearly better prognosis than lymph node-positive TNBC patients. This result was in accordance with other studies [20–22]. It is well known that in breast cancer, the number of positive lymph nodes is inversely associated with prognosis and survival. However, Hernandez-Aya LF *et al.* [18] found that the prognosis of TNBC may not be affected by the number of positive lymph nodes. In our study, a higher number of positive lymph nodes did not completely guarantee worse outcomes. Instead, worse BCSS or OS with increasing lymph node involvement was not observed until a cut-off value of ten metastatic lymph nodes. Although TNBC is an aggressive disease, hematogenous metastasis is significantly more frequent than nodal metastasis in TNBC. Accordingly, we recognized that its prognosis is driven in part by the biology of triple-negative disease, as well as by clinical variables such as the extent of nodal involvement upon surpassing a cut-off value. Our findings might have an effect on clinical practice for TNBC patients. In particular, our study reinforced that N3 patients had substantial risk of mortality due to breast cancer and all causes; thus, N3 patients should be treated with aggressive systemic and locoregional therapy, especially intensive radiation therapy, which was essential for lymph node-positive patients.

In addition, tumor size was consistently recognized as another reliable factor confounding the prediction of outcomes among women with TNBC [23]. Our study showed that nodal status and tumor size exhibited distinct influences on prognosis. Further analysing the causes of this disparity, we initially suspected that tumor size had a great effect on the relationship between nodal status and prognosis. However, it has been reported by Carter *et al.* [19] that survival outcomes worsened with increased lymph node involvement regardless of tumor size. Therefore, we considered another potential explanation: as demonstrated by previous analyses of patients with TNBC, tumor size and lymph node status may not linearly correlate with survival outcomes [1, 24]. Wo *et al.* [25] reported that among ER-negative patients with four or more positive lymph nodes, those with T1b tumor stage exhibited a significantly lower rate of breast cancer–specific mortality (BCSM) than those with T1a tumor stage; however, in that study, there was no significant difference in BCSM between patients with T1a and either T1c or T2 tumor stages. Therefore, further analysis of the relationship between tumor size and nodal status is required in the future.

Our study contains several limitations. In terms of follow-up data, it is well known that information regarding HER-2 expression in the SEER database was not available until 2010. We were therefore compelled to focus on the short-term survival outcomes after initial diagnosis and to identify any outcome-related factors and an inadequate follow-up duration may lead to skewed results. But for TNBC subtype, an early peak of recurrence occurs within the first 2-3 years after diagnosis. In addition, differences in treatments could influence survival outcomes, but information regarding adjuvant or neoadjuvant chemotherapy is not available in the SEER database.

In conclusion, our study revealed that among all TNBC patients, those with N0 lymph node status experienced significantly better BCSS and OS than those with N1–N3 lymph node status. Additionally, for patients with T1 or T3, the prognosis of the N1 group was better than that of the N3 group but appeared similar to that of the N2 group. Therefore, nodal status and tumor size exhibited distinct interaction patterns for predicting the outcomes of TNBC. These results provide deeper insight into the prognostic value of nodal status for TNBC.

## MATERIALS AND METHODS

### Ethics statement

Our study was approved by an independent ethics committee/institutional review board at Fudan University Shanghai Cancer Center (Shanghai Cancer Center Ethics Committee). The data in the SEER database do not require informed patient consent because cancer is a disease reported by every state of the United States.

### Patients

We used SEER*Stat version 8.2.1 to generate a case list. We identified 10,771 patients according to the following inclusion criteria: female; year of diagnosis from 2010 to 2012; age of diagnosis between 20 years and 74 years; breast cancer as the first and only malignant cancer diagnosis; pathologically confirmed infiltrating duct carcinoma-not otherwise specified (IDC-NOS, ICD-O-3 8500/3) or lobular carcinoma-not otherwise specified (ILC-NOS, ICD-O-3 8520/3); unilateral cancer; TNBC subtype (absence of ER, PR, and HER2); histological grades I-III; AJCC stages I-III; known tumor size category; and known lymph node status. We excluded patients with inflammatory breast cancer, in situ disease, histological grade IV (SEER program code: undifferentiated or anaplastic), and no record of surgery type or radiation therapy. We calculated follow-up durations from January 1, 2010 to December 31, 2012.

Patients were categorized according to their tumor size, i.e., T1 (tumor size ≤2 cm), T2 (tumor size 2-5 cm) or T3 (tumor size >5 cm), and lymph node status. The number of positive lymph nodes was categorized into one of four groups: N0 (no positive lymph nodes), N1 (1-3 positive lymph nodes), N2 (4-9 positive lymph nodes), and N3 (≥10 positive lymph nodes).

### Statistical analyses

The demographic and clinical characteristics of the included cases were compared between the four lymph node groups using a Chi-squared test. The Kaplan-Meier method was performed to generate survival curves, and the log-rank test was performed to compare the unadjusted BCSS and OS rates of patients with different lymph node status. BCSS was measured from the date of diagnosis to the date of breast cancer death. OS was defined as the time from the date of diagnosis to the date of death due to any cause (including breast cancer) or the last follow-up. In addition, we conducted pairwise comparisons using the Sidak adjustment method and found that the N1 group was the only group that exhibited a significant difference in prognosis. Accordingly, we used N1 as a reference to compare the different HRs of BCSS and OS within each tumor size category. Adjusted HRs with 95% CIs were calculated using a Cox proportional hazard regression model to estimate the outcome-related factors. All tests were two-tailed. P-values <0.05 were considered significant. All statistical analyses were performed utilising SPSS version 20.0 software (IBM SPSS Statistics, Chicago, IL, US).

## SUPPLEMENTARY TABLE


