# Supplementary material for: Effect of nodal status on clinical outcomes of triple-negative breast cancer: a population-based study using the SEER 18 database
Source: Oncotarget. 2016 May 18;7(29):46636–45. doi: 10.18632/oncotarget.9432 (PMC5216824; doi:10.18632/oncotarget.9432)
Supplement: Supplementary file 1 [file oncotarget-07-46636-s001.doc]

**Supplementary Table S1:** Univariate Cox proportional hazard model.

| **Variable** | **BCSS** | | |  | **OS** | | |
| --- | --- | --- | --- | --- | --- | --- | --- |
|  | **HR** | **95% CI** | **P ^c^** |  | **HR** | **95% CI** | **P ^c^** |
| **Age (years)** |  |  | **0.017** |  |  |  | 0.834 |
| **≥50** | 1 |  |  |  | 1 |  |  |
| **<50** | 1.307 | 1.048-1.629 |  |  | 0.981 | 0.819-1.175 | **0.006** |
| **Race** |  |  | **0.038** |  |  |  | **0.002** |
| **White** | 1 |  |  |  | 1 |  |  |
| **Black** | 1.259 | 0.981-1.616 |  |  | 1.254 | 1.031-1.525 |  |
| **Other ^a^** | 0.659 | 0.391-1.110 |  |  | 0.590 | 0.384-0.906 |  |
| **Marital status** |  |  | **0.006** |  |  |  | **<0.001** |
| **Married** | 1 |  |  |  | 1 |  |  |
| **Not married ^b^** | 1.352 | 1.091-1.676 |  |  | 1.485 | 1.256-1.756 |  |
| **Laterality** |  |  | 0.240 |  |  |  | 0.276 |
| **Left** | 1 |  |  |  | 1 |  |  |
| **Right** | 0.879 | 0.710-1.090 |  |  | 0.911 | 0.770-1.078 |  |
| **Histological type** |  |  | 0.725 |  |  |  | 0.058 |
| **Infiltrating duct carcinoma** | 1 |  |  |  | 1 |  |  |
| **Lobular carcinoma** | 1.193 | 1.445-3.198 |  |  | 1.830 | 0.979-3.422 |  |
| **Grade** |  |  | **<0.001** |  |  |  | **0.014** |
| **III** | 1 |  |  |  | 1 |  |  |
| **I/II** | 0.530 | 0.374-0.753 |  |  | 0.738 | 0.579-0.941 |  |
| **Tumor size** |  |  | **<0.001** |  |  |  | **<0.001** |
| **T1** | 1 |  |  |  | 1 |  |  |
| **T2** | 2.758 | 2.109-3.608 |  |  | 2.178 | 1.787-2.654 |  |
| **T3** | 7.758 | 5.681-10.593 |  |  | 5.385 | 4.226-6.861 |  |
| **Lymph node status** |  |  | **<0.001** |  |  |  | **<0.001** |
| **N0** | 1 |  |  |  | 1 |  |  |
| **N1** | 3.692 | 2.872-4.745 |  |  | 2.461 | 2.025-2.992 |  |
| **N2** | 4.300 | 2.979-6.205 |  |  | 3.493 | 2.645-4.614 |  |
| **N3** | 11.377 | 8.231-15.725 |  |  | 7.440 | 5.719-9.678 |  |
| **Type of surgery** |  |  | **<0.001** |  |  |  | **<0.001** |
| **Breast-conserving surgery** | 1 |  |  |  | 1 |  |  |
| **Mastectomy** | 2.210 | 1.660-2.658 |  |  | 2.033 | 1.691-2.445 |  |
| **None** | 6.862 | 4.570-10.304 |  |  | 7.001 | 5.132-9.552 |  |
| **Radiation therapy** |  |  | **0.004** |  |  |  | **<0.001** |
| **No** | 1 |  |  |  | 1 |  |  |
| **Yes** | 0.728 | 0.587-0.903 |  |  | 0.603 | 0.507-0.716 |  |

Abbreviation: CI, [confidence](javascript:void(0);) [interval](javascript:void(0);).

^a^ Other includes American Indian/native Alaskan and Asian/Paciﬁc Islander.

^b^ Not married includes divorced, separated, single (never married), unmarried or domestic partner and widowed.

^c^ P values were calculated among all groups using a univariate Cox proportional hazard regression model, and bold type indicates significance.
